# Visual attention and role recognition in bullying vignettes in preadolescents and adults

**DOI:** 10.1111/bjep.70039

**Published:** 2025-10-05

**Authors:** Laura Menabò, Annalisa Guarini

**Affiliations:** ^1^ Department of Psychology “Renzo Canestrari” University of Bologna Bologna Italy

**Keywords:** age differences, bullying roles, eye‐tracking, social information processing, visual attention

## Abstract

**Background:**

Bullying research has traditionally relied on self‐reported measures such as questionnaires and interviews. Previous studies have shown developmental differences in attention mechanisms, with adults relying more on top‐down processing and younger individuals on bottom‐up attention. However, it remains unclear whether these differences extend to bullying scene observation and how they influence the perception of different bullying roles.

**Aims:**

This study examined differences in visual attention (total fixation duration, visit count, fixation count) and verbal recognition of bullying roles between preadolescents and adults.

**Sample:**

The study included 80 participants: 37 preadolescents (*M*_age_ = 10.11, SD = 1.10) and 43 adults (*M*_age_ = 30.72, SD = 4.89).

**Methods:**

Participants viewed 12 vignette‐based bullying scenes while their eye movements were recorded using an eye tracker. They then provided verbal descriptions of each observed vignette.

**Results:**

Both groups primarily fixated on bullies and victims, reinforcing the centrality of the bully–victim dyad. However, adults allocated significantly more attention to the other roles and described them with greater accuracy than preadolescents. In particular, in adults, defenders and pro‐bullies attracted more fixations, visits, and total fixation time, while bystanders received more total fixation time.

**Conclusions:**

The findings suggest that adults process bullying situations in a more structured and holistic manner, likely due to top‐down attentional mechanisms shaped by social experience. These developmental differences highlight the crucial role of adults in fostering awareness of all bullying roles among preadolescents, emphasizing the need for interventions that encourage broader role recognition beyond the bully–victim dynamic.

## INTRODUCTION

According to the comprehensive approach to mental health adopted by the European Commission in 2023, bullying is acknowledged as a significant factor in negatively impacting children's and young people's mental health, potentially leading to long‐term psychological issues and suicide in the most severe cases (Holt et al., 2015). Over the years, bullying research has mainly relied on traditional methods such as questionnaires, interviews, and focus groups to gather information on individuals' experiences, perceptions, and attitudes towards bullying (Smith et al., 2021). These methods have provided valuable insights into the phenomenon; however, they are mostly based on self‐reported data, which may be influenced by memory biases or social desirability (Hunter et al., 2021; Maran & Begotti, 2021). In recent years, a growing body of research has aimed to complement traditional approaches by incorporating objective behavioural measures, such as eye‐tracking technology, to gain a deeper understanding of how individuals visually process bullying situations (Caravita et al., 2016; McConnell & Troop‐Gordon, 2021; Menabò et al., 2024, 2025; Troop‐Gordon et al., 2019).

One theoretical framework that helps to explain how attention is allocated in bullying situations is the Social Information Processing (SIP) model (Crick & Dodge, 1994). According to the SIP, people follow a series of cognitive steps that shape their behaviour in social situations. Specifically, this process includes: (1) encoding social cues, (2) interpreting these cues, (3) identifying goals, (4) generating possible responses, (5) selecting a response, and (6) executing the chosen behaviour (Crick & Dodge, 1994). In particular, the initial encoding is crucial in shaping how individuals process social interactions, including bullying situations (e.g., Camodeca et al., 2003). This phase determines how information is interpreted, ultimately influencing decision‐making and behavioural responses (Garon et al., 2018). When social cues receive greater attention during encoding, they are more likely to be processed effectively and recognized later. In recent years, some studies have examined the first two steps of the SIP model in the broader context of aggression (e.g., Horsley et al., 2010; Troop‐Gordon et al., 2018) and in specific bullying situations (Caravita et al., 2016; Menabò et al., 2024, 2025; Troop‐Gordon et al., 2019), using eye‐tracker technology to analyse attention patterns. These studies have yielded interesting new findings, which are described in the following sections. However, none of these studies have focused on how such processes might vary across developmental stages. Indeed, attention allocation is also influenced by developmental stage, as the ability to detect and interpret complex social cues develops progressively over time, in parallel with the maturation of specific brain regions involved in social perception and attentional regulation (Walbrin et al., 2020). In addition, the accumulation of direct and indirect interaction experiences can influence attention processes (Mihai, 2022).

This study addresses this gap by combining eye‐tracking data, which capture implicit attentional processes, with narrative descriptions, which reflect explicit cognitive interpretation, in order to explore how age affects both the visual and verbal recognition of bullying roles.

### Development of social attention and age‐related differences

Age‐related differences in attentional processes have been primarily investigated through non‐social visual tasks, such as visual search or processing isolated visual stimuli. These studies have highlighted a fundamental distinction between two attentional mechanisms: bottom‐up attention, driven by perceptually salient external stimuli, and top‐down attention, guided by prior knowledge, cognitive strategies, and internalized schemas (Mancas, 2009; Vuilleumier et al., 2001). Research has shown that children rely more heavily on bottom‐up processes, tending to focus on visually striking features such as colour movement, and emotionally salient stimuli (Açık et al., 2010), whereas with age, individuals show a growing ability to engage top‐down strategies, allowing adults to direct their gaze more selectively and purposefully toward task‐relevant information (Donnelly et al., 2007).

Top‐down and bottom‐up processes were particularly relevant in interpreting dynamic interactions that required the ability to integrate multiple sources of information, infer intentions, and roles that are often implicit (Capozzi & Ristic, 2020; Quadflieg & Penton‐Voak, 2017). This capacity develops gradually and improves alongside the maturation of executive control over attention, which continues through adolescence (Amso & Scerif, 2015; Luna et al., 2008). Voluntary gaze control and attentional flexibility, essential to shifting from bottom‐up to more sophisticated top‐down processing, depend on brain networks involved in social cognition, such as the posterior superior temporal sulcus (pSTS), the temporoparietal junction (TPJ), and the medial pre‐frontal cortex (mPFC), which are not fully developed until late childhood or early adulthood (Richardson et al., 2018; Walbrin et al., 2020).

In parallel with neurocognitive development, social attention is also refined through the accumulation of direct and indirect interaction experiences. Frequent exposure to social situations contributes to the construction of more complex interpretive schemas that guide the observation and interpretation of social cues (Quadflieg & Westmoreland, 2019; Ricciardelli, 2001). Such experiences enrich an individual's ‘social vocabulary’, enabling the recognition of different interactions and facilitating their integration into a coherent representation of the event. In this sense, attentional development is not solely the result of biological maturation but also reflects a cumulative learning process, in which social experience provides the framework for modulating the allocation of attentional resources and the subsequent interpretation of the observed scene (Mihai, 2022).

Despite the evident developmental relevance of attention to social interactions, very few studies have directly compared adults and younger individuals in tasks involving the observation of interacting figures, suggesting differences. For example, De Lillo et al. (2021) found that young adults spent more time looking at their conversation partner's face and engaging visually with the people around them, whereas adolescents focused more on non‐social but perceptually salient elements, such as nearby objects. In this case, the interaction involved only two actors, and the results aligned with the predominance of bottom‐up processes in younger participants and more effective top‐down modulation in adults. In another study, Mihai (2022) found that children spent more time looking at the background than adults in complex scenes with three and four people. This greater exploration of the background may indicate that children need more contextual information to process complex situations or that they have a reduced ability to filter out irrelevant stimuli when presented with numerous visual elements. Overall, these findings indicate that the ability to strategically and selectively attend to important cues in social interactions develops with age. Yet, no study to date has directly compared how different age groups attend to and interpret bullying roles, leaving an important gap in our understanding of developmental differences in the perception of bullying dynamics.

### Attention in bullying and social roles

To gain deeper insight into the attentional dynamics of bullying, recent studies have employed eye‐tracking to analyse how students encode social cues in bullying situations. Troop‐Gordon et al. (2019) examined lower secondary school students' gaze patterns when watching bullying video clips that included the bully, victim, pro‐bully, defender, and bystander roles. Their findings showed that victimization was associated with high levels of aggressiveness for moderate or high levels of attention to bullying. Furthermore, aggressiveness, regardless of the level of victimization, was negatively correlated with attention to the victim. Extending this work, McConnell and Troop‐Gordon (2021) showed that students who both experienced high victimization and focused on the bully were more likely to engage in retaliatory behaviours than those with high victimization but without this attentional bias.

A recent line of research has been carried out by Menabò et al., analysing attention patterns in the exploration of different roles in bullying. In one study, Menabò et al. (2024) showed that lower secondary‐school students with experiences of both victimization and perpetration directed more attention to the bully and pro‐bully roles when observing vignettes depicting all roles (bully, victim, pro‐bully, defender, bystander). In another study, Menabò et al. (2025) found that middle‐school students focused most of their visual and verbal attention on the bully and the victim, while defenders, pro‐bullies, and passive bystanders were noticed far less. These results confirm a representation of bullying that is almost exclusively centered on the bully–victim dyad, as already highlighted by other methods such as drawings (Bosacki et al., 2006) and questionnaires (Guarini et al., 2019).

With adults, however, knowledge is still limited regarding how they encode social cues in bullying situations. Overall, studies with adults (e.g., parents, teachers) have focused on their knowledge in identifying the core elements of the bullying phenomenon (Stives et al., 2023), while other studies have explored the perceived severity of different forms of bullying, as well as consequences (Bauman & Del Rio, 2006; Yoon et al., 2016). The only exception is the study by Caravita et al. (2016), which explored differences in attentional allocation across bullying and cyberbullying versus prosocial and neutral interactions, considering young adults' retrospective experiences of victimization. The results showed that individuals with victimization experiences tended to divert their early attention away from bullying and cyberbullying scenes. However, this study considered different types of interactions without attention to the distinct roles within bullying situations, and to our knowledge, no study has used eye‐tracking to analyse how adults observe the different roles.

Yet studying the roles in bullying is essential. As highlighted by the Social Information Processing (SIP; Crick & Dodge, 1994), attention to social stimuli represents a fundamental step for interpreting interactions and guiding behavioural responses. Research has shown that less salient roles, such as defenders, pro‐bullies, and bystanders, play a pivotal part in sustaining or counteracting aggression (Gini et al., 2021). When these roles are overlooked, however, the understanding of the collective becomes limited, reducing opportunities for effective educational and preventive interventions (Haataja et al., 2015). Conversely, attending to all roles allows for a more comprehensive view of the relational processes that reinforce or disrupt aggression, providing a stronger basis for targeted interventions. For this reason, it is important to examine how both preadolescents and adults visually process bullying scenes. To date, however, no study has analysed adults' attentional responses to the different roles involved and how these responses vary across developmental stages. Investigating how attention to bullying stimuli is distributed across age groups, including adulthood, would not only deepen our understanding of the developmental trajectory of attentional dynamics but also yield critical insights for enhancing prevention strategies and educational practices.

## AIMS

This study aimed to investigate differences between preadolescents and adults in observing bullying vignettes. We analysed differences in the perception of different roles (the bully, the victim, the defender, the pro‐bully, the bystander), investigating attentional indices (total fixation duration, visit count, fixation count) and collecting verbal descriptions of the different roles included in the vignettes. This approach allowed us to compare implicit visual attention patterns captured by the eye‐tracker with explicit descriptions in participants' words. Eye‐tracking attentional indices provide insights into implicit processes, such as automatic attentional responses, which are not accessible through introspection or self‐reporting. On the other hand, verbal descriptions reflect explicit cognitive processes—conscious thoughts participants are aware of and can articulate (DeCoster et al., 2006). By combining these implicit and explicit measures, we aimed to gain a deeper understanding of participants' awareness and different perceptions of the roles involved in bullying.

Based on prior research with middle‐school students (Menabò et al., 2025), we expected both groups to focus primarily on the bully and the victim, confirming the centrality of the bully–victim dyad. However, we hypothesized age‐related differences for the secondary roles (defenders, pro‐bullies, passive bystanders). Specifically, we expected that adults, thanks to the cognitive development of attentional control and their broader social experience, would implicitly consider secondary roles as more relevant, showing stronger attentional indices towards them. This expectation aligns with findings from broader research on social interaction, which shows that individuals become better at selectively attending to socially meaningful cues with age (De Lillo et al., 2021; Mihai, 2022). In addition, we anticipated that adults would also mention these roles more frequently in their verbal descriptions, confirming at an explicit level a greater awareness of the complexity of bullying dynamics. As suggested by a recent constructivist perspective (Rowland et al., 2025), this broader linguistic production can emerge from the interplay between cognitive maturation and life experiences. While the development of attentional control and symbolic systems enables more complex representations, the communicative environment itself adapts and expands with age (Rowland et al., 2025). This process is evident in the development of pragmatic skills (Garaffa & Mazzaggio, 2025), where the interaction between brain maturation and environmental factors plays a particularly important role in shaping the use of language in social contexts (Garaffa & Mazzaggio, 2025). Thus, adults' greater propensity to identify and verbalize secondary roles can be seen as the outcome of a continuous, reciprocal process between cognitive development and environmental input, which progressively enriches the ways in which social dynamics are observed and described.

## METHOD

### Participants

The sample consisted of 84 participants divided into two groups: 40 students from the 5th grade of primary school to the 6th grade of middle school and 44 adults. In line with Menabò et al. (2024), participants were excluded if at least one index (total fixation duration, visit count, and fixation count; for their definition, see the paragraph ‘Eye‐tracker Indices and Interview Coding’) deviated by ±2 standard deviations. The final sample consisted of 80 participants: 37 students (*M*_age_ = 10.11, SD_age_ = 1.10) and 43 adults (*M*_age_ = 30.72, SD_age_ = 4.89). In terms of gender distribution, the student group included 16 males (43%) and 21 females (57%), while in the adult group, 13 (30%) were males and 30 (70%) were females. All the participants were Italian.

The recruitment of school participants involved one class from one public primary school (fifth grade) and one class from one public lower secondary school (sixth grade) in the Emilia‐Romagna region, Northern Italy. Across the two classes, two parents from the primary school did not provide consent, resulting in a participation rate of 90% for the primary school and 100% for the lower secondary school. Adult participants were recruited through personal networks.

The study adhered to ethical guidelines for the protection of human participants, in compliance with Italian legal requirements, and was approved by the Bioethics Committee of the authors' university. Informed consent was obtained from the parents of all participating students, while adult participants signed their own consent forms. Additionally, students were verbally informed about their participation in the experiment and were assured that their decision to participate or not would have no consequences. No economic incentives were provided to participants.

### Stimuli and apparatus

Stimuli for the eye‐tracking task were composed of 12 vignette drawings representing different types of bullying episodes (three for physical bullying, three for verbal bullying, three for relational bullying, and three for cyberbullying). All roles (bully, victim, pro‐bully, defender, and bystander) were represented in each scene, and all the vignettes were gender‐balanced (for each type of bullying, one vignette represented only males, one only females, and one was mixed‐gender; for more information on the stimuli, please refer to Menabò et al., 2025). The drawings were displayed on a 19‐inch monitor at 1600 × 900 pixels resolution, and the Tobii Pro X2/60 recorded participants' eye movements, sampling gaze location at 60 Hz.

To ensure that attentional differences were not driven by visual properties or spatial layout, we conducted three control analyses. First, we examined perceptual salience using the spectral residual approach in Python. Saliency maps were generated for each vignette, and we calculated the mean pixel salience within the predefined AOIs corresponding to each role (see ‘Eye‐tracker Indices and Interview Coding’ paragraph for details). This method highlights visually prominent regions based on image properties such as contrast, colour, and texture. A repeated‐measures ANOVA revealed no significant differences in perceptual salience across roles, *F* (4, 50) = .14, *p* = .968, *η* ^2^ = .01, indicating that no role was inherently more visually striking than the others.

Second, we assessed absolute spatial centrality, defined as the angular distance of each role from the geometric center of the vignette. This analysis tested whether some roles were systematically positioned closer to the visual center, where viewers' gaze is naturally more likely to land. Again, no significant differences emerged, *F* (4, 44) = 2.05, *p* = .157, *η* ^2^ = .14, suggesting that absolute positioning did not bias attentional patterns.

Finally, we evaluated relational spatial proximity, defined as the angular distance (in degrees of visual angle) between each pair of roles. For each role, we computed its mean distance from all others in the same vignette, capturing how spatially integrated it was within the overall configuration of characters. A repeated‐measures ANOVA showed a significant main effect of role, *F* (4, 44) = 11.48, *p* < .001, *η* ^2^ = .44. However, Bonferroni‐corrected comparisons revealed no consistent pattern across vignettes. This discrepancy suggests that the global effect detected by the ANOVA reflects variability across vignettes, introduced partly by design to preserve ecological validity, rather than systematic differences between specific roles. In other words, while some roles were occasionally positioned closer to or farther from others, this variability was inconsistent and did not produce stable pairwise differences. Thus, relational positioning was unlikely to confound the observed attentional differences.

Together, these three controls indicate that the observed attentional differences cannot be attributed to perceptual salience, absolute centrality, or relational proximity among roles.

### Procedure

For the student participants, the experiment was carried out at their schools in a designated room by the first author, assisted by a psychologist specializing in bullying and cyberbullying. After being seated at a table in front of the screen, students were informed that their eye movements would be tracked as they viewed scenes depicting different types of bullying. The eye‐tracker was then calibrated and validated to ensure a gaze accuracy of .50 degrees or better. Students could control the pace of the presentation by pressing the right arrow key to move to the next image. Once the experiment was completed, the vignettes were shown again on the screen without eye tracking, and students were asked to describe what they had observed in each scene briefly. To avoid influencing their responses, the researcher avoided referencing specific roles or asking leading questions. Each student's descriptions were recorded verbatim. For adult participants, the procedure was identical, though the experiment took place in the Developmental Psychology Lab at the University of Bologna. The entire procedure lasted approximately 10 minutes per participant.

### Eye‐tracker indices and interview coding

To extract the relevant indices, we defined each role in the scene as a separate area of interest (AOI). An AOI represents a specific region within a stimulus where attention‐related metrics can be measured. Each vignette, therefore, contained five distinct AOIs corresponding to the roles depicted: bully, victim, pro‐bully, defender, and bystander. Once the AOIs were established, we extracted the following measures for each AOI: total fixation duration (measured in seconds, representing the cumulative time spent fixating within an AOI), visit counts (the number of times an AOI was visited), and fixation counts (the number of times a participant fixated within an AOI).

The interview data were coded using the same content analysis used by Menabò et al. (2025). Each role depicted in the vignettes (bully, victim, pro‐bully, defender, passive bystander) was coded separately within each narrative description. This means that for every vignette, each role received an individual score based on whether it was mentioned and how accurately it was described, rather than assigning a single overall score to the entire narrative. Each role in the vignettes was assigned to one of the following categories: Category 0 if not mentioned, Category 1 if present and accurately described, and Category 2 if present but inaccurately described.

We reported three examples of vignette descriptions, each assigned a score of 0, 1, or 2 based on the accuracy of role identification. The following examples were reported by preadolescents.

Example 1: *‘One child pushes another. One laughs, one remains passive, and another helps the fallen child get up’ (English translation)*. In this example, all roles were correctly mentioned and identified (score = 1 for each role).

Example 2: *‘A girl attempts to push another’ (English translation)*. Here, only the bully and the victim were correctly identified (score = 1 for each role), while the pro‐bully, the defender, and the bystanders were not mentioned (score = 0 for each role).

Example 3: *‘One child pushes another. Two friends of the one who pushed him laugh, while another child attempts to help the victim stand up’ (English translation)*. In this example, the bully, the victim, the pro‐bully, and the defender were correctly identified (score = 1 for each role). However, the bystander was mistakenly categorized as a pro‐bully (score = 2).

We also reported three examples of descriptions produced by adults.

Example 1: *‘A boy pushes another boy. One observer laughs, another remains indifferent, while a third, who is a friend of the fallen boy, tries to help him up’ (English translation)*. In this example, all roles were correctly mentioned and identified (score = 1 for each role).

Example 2: *‘A girl trips another girl. One bystander appears uninterested, while another expresses indignation’ (English translation)*. In this case, the bully, the victim, the defender, and the bystander were correctly identified (score = 1 for each role), while the pro‐bully was not mentioned (score = 0).

Example 3: *‘There is a girl with a smartphone who teases a little boy who fell while her friend is laughing. The boy is helped by a classmate. And one student looks disapprovingly at the bully’ (English translation)*. Here, the bully, the victim, the pro‐bully, and the defender are correctly identified (score = 1 for each role). However, the bystander is misidentified as a defender (score = 2).

The first author conducted the initial analysis and coding of all interviews, while 20% of the interviews were randomly selected and independently coded by the last author. Intercoder reliability was assessed using Cohen's kappa, yielding perfect agreement (κ = 1) for the bully and the victim. Reliability scores for the other roles were also strong, with κ = .85 for the defender, κ = .83 for the bystander, and κ = .80 for the pro‐bully.

### Data analysis

To analyse differences in eye movements, we first calculated the average value of total fixation duration, visit count, and fixation count for each role. We then conducted three mixed ANOVAs to examine the attentional indices separately. In each ANOVA, the within‐subject factor was the role (bully, victim, pro‐bully, defender, and bystander), and the between‐subject factor was age, divided into two groups: preadolescents and adults. The mixed ANOVAs were performed using a Type III sum of squares method, which is robust for handling unbalanced designs. We applied Greenhouse–Geisser corrections to account for potential violations of sphericity where necessary, and we also calculated eta‐squared (*η* ^2^) to measure effect size. Eta‐squared values range from 0 to 1, with values below .01 indicating a negligible effect, .01 to .06 indicating a small effect, .06 to .14 indicating a moderate effect, and values above .14 indicating a large effect (Cohen, 1988).

To analyse differences in the verbal descriptions, we performed separate Chi‐Square (χ^2^) tests of independence for the description of each role within any vignette. Yates' correction for continuity was applied to cells with values below 5. To interpret the results, we examined the standardized residuals to measure the difference between observed and expected counts. A standardized residual greater than or less than 2 indicates a notable deviation from expected frequencies under the null hypothesis (Naioti & Mudrak, 2022). When the Chi‐Square tests showed significant associations, we calculated Cramer's V to determine the strength of the associations. Cramer's V ranges from 0 to 1, where values from 0 to .1 indicate a weak or negligible association, .1 to .3 indicate a moderate association, .3 to .5 indicate a relatively strong association, and values above .5 indicate a strong association (Akoglu, 2018). All the analyses were performed using R software (version 4.4.0).

## RESULTS

### Attentional indices analysis

**FIGURE 1:**
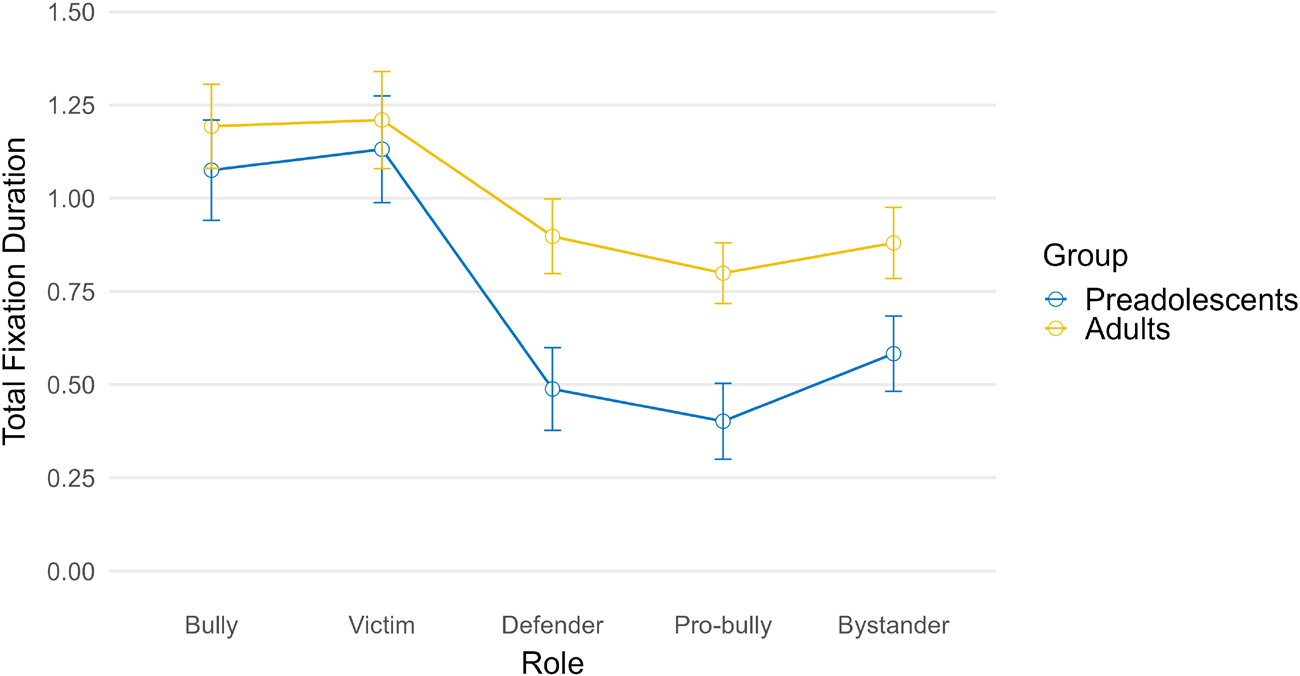
Interaction of age group and role on total fixation duration in bullying vignettes.

The mixed ANOVA on total fixation duration revealed a significant within‐subject effect for roles, *F* (3.47, 270.98) = 37.50, *p* < .001, *η* ^2^ = .325 (large effect), indicating differences depending on the observed role. Specifically, post hoc tests with Bonferroni adjustments revealed that the bully and the victims were fixated longer than others. While the between‐subject effect for age was not significant, *F* (1,78) = 3.53, *p* = .064, *η* ^2^ = .018, the interaction effect between role and age was significant, *F* (3.47, 270.98) = 3.17, *p* = .019, *η* ^2^ = .039 (small effect), suggesting that the impact of roles on fixation duration varied across age groups. Indeed, while no significant differences were found for the bully role (*p* = .501) and the victim (*p* = .686), adults observed the defender (*p* = .008), the pro‐bully (*p* = .003), and the bystander (*p* = .042) significantly longer than younger participants (Figure 1).

**FIGURE 2:**
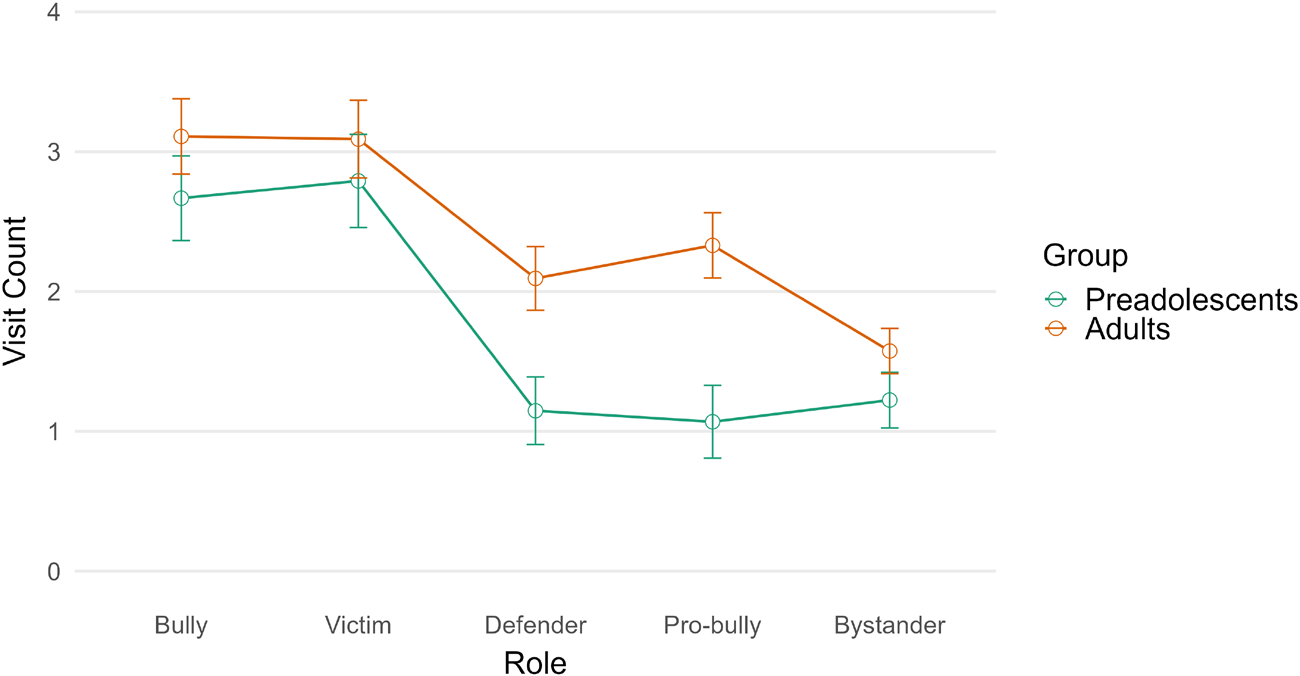
Interaction of age group and role on visit count in bullying vignettes.

The mixed ANOVA on visit count revealed a significant within‐subject effect, *F* (3.51, 273.74) = 53.51, *p* < .001, *η* ^2^ = .407 (large effect). Post‐hoc tests with Bonferroni adjustments indicated that the bully and the victim received a significantly higher number of visit counts compared to the other roles. The between‐subject effect for age was significant, *F* (1, 78) = 4.54, *p* = .040, *η* ^2^ = .055 (small effect), highlighting that adults gave overall more visits compared to the preadolescents (*M* = 2.44, *SD *= 1.42 and *M* = 1.78, *SD *= 1.34, respectively). The interaction effect between role and age was also significant, *F* (3.51, 273.74) = 4.35, *p* = .003, *η* ^2^ = .053 (small effect). Analysing the interaction effect, no significant differences emerged for the bully (*p* = .277), the victim (*p* = .490), and the bystander (*p* = .170). By contrast, adults visited the defender (*p* = .006) and the pro‐bully (*p* < .001) more frequently than younger participants (Figure 2).

**FIGURE 3:**
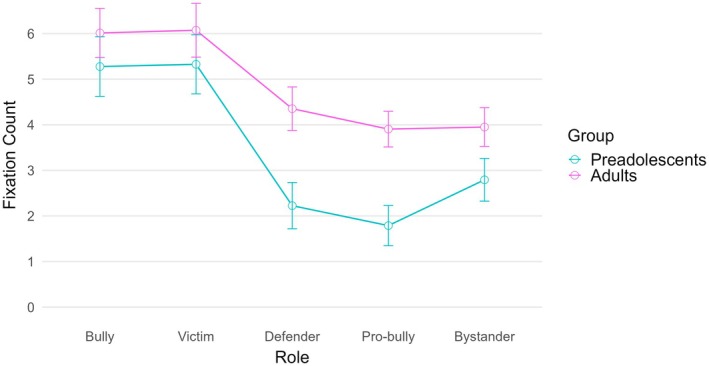
Interaction of age group and role on fixation count in bullying vignettes.

The mixed ANOVA on fixation count showed a significant within‐subject effect, *F* (3.55, 276.87) = 46.01, *p* < .001, *η* ^2^ = .371 (large effect). As happened for the total fixation duration and the visit count, post‐hoc tests with Bonferroni adjustments showed that the bully and the victim significantly received more fixation compared to the other roles. The between‐subject effect for age was also significant, *F* (1, 78) = 4.66, *p* = .034, *η* ^2^ = .056 (small effect), suggesting adults gave more fixations compared to the younger group (*M* = 4.86, SD = 2.90 and *M* = 3.48, SD = 2.78 respectively). Finally, the interaction effect between role and age was also significant, *F* (3.55, 276.87) = 2.94, *p* = .026, *η* ^2^ = .036 (small effect). No significant differences were observed for the bully (*p* = .384), the victim (*p* = .396), or the bystander (*p* = .070), while adults made more fixations on the defender (*p* = .003) and the pro‐bully (*p* < .001) compared to younger participants (Figure 3).

Descriptive statistics for attentional measures across all roles in both preadolescents and adults are presented in Table 1.

**TABLE 1:** Means and standard deviations for total fixation duration, visit count, and fixation count among roles in preadolescents and adults.

Index	Group	Bully		Victim		Defender		Pro‐bully		Bystander	
		*M*	SD	*M*	SD	*M*	SD	*M*	SD	*M*	SD
Total fixation duration	Preadolescents	1.06	.82	1.13	.87	.49	.67	.40	.62	.58	.62
	Adults	1.19	.74	1.21	.86	.90	.66	.80	.53	.88	.63
Visit count	Preadolescents	2.67	1.84	2.79	2.03	1.15	1.48	1.06	1.58	1.22	1.21
	Adults	3.11	1.76	3.09	1.82	2.09	1.49	2.33	1.54	1.57	1.06
Fixation count	Preadolescents	5.28	3.98	5.33	3.94	2.22	3.08	1.79	2.68	2.80	2.85
	Adults	6.01	3.54	6.07	3.97	4.35	3.13	3.91	2.57	3.95	2.78

### Interview coding analysis

Regarding the verbal description, the analyses conducted for the bully role indicated a statistically significant association *χ* ^2^ (2) = 6.71, *p* = .035 with a Cramér's *V* of .081 (small effect). As standardized residuals were not greater than 2, the pattern was consistent across age groups and no single category strongly deviated from expected counts (Table 2). For the victim role, no statistically significant association emerged, *χ* ^2^ (2) = 4.27, *p* = .118 (Table 2).

**TABLE 2:** Frequency distribution and percentages of role recognition in preadolescents and adults and chi‐squared test and Cramér's *V* results.

Role	Group	Absent		Correct		Incorrect		*X* ^2^	Cramér's *V*
		*n*	%	*n*	%	*n*	%		
Bully	Preadolescents	32	6	471	94	0	0	6.71*	.081
	Adults	23	4	498	95	5	1		
Victim	Preadolescents	33	7	469	93	1	0	4.27	.064
	Adults	20	4	504	96	2	0		
Defender	Preadolescents	270	54	198	39	35	7	204.46***	.446
	Adults	66	13	422	80	36	7		
Pro‐bully	Preadolescents	175	35	321	64	7	1	94.77***	.304
	Adults	52	10	469	89	4	1		
Bystander	Preadolescents	337	67	144	29	22	4	362.47***	.594
	Adults	57	11	455	87	13	2		

*Note*: The role recognition categories are defined as follows: Absent (Category 0): the role was not mentioned. Correct (Category 1): the role was identified and accurately described. Incorrect (Category 2): the role was mentioned but inaccurately described.

*p < .05; ***p < .001.

The defender role presented a highly significant association with age group, *χ* ^2^ (2) = 204.46, *p* < .001, with a Cramér's *V* of .446 (strong effect). The standardized residual showed that preadolescents significantly omitted the defender role compared to adults (*ASR* = 8.2) and that adults were significantly more likely to correctly name and describe the defender role (*ASR* = 5.9, Table 2).

In the case of the pro‐bully role, the association with age group was significant, *χ* ^2^ (2) = 94.77, *p* < .001, with a Cramér's *V* of .304 (moderate effect). The standardized residual indicated that preadolescents were significantly more likely to omit the pro‐bully (*ASR* = 6.1) than adults, while adults were significantly more likely to correctly name and describe it (*ASR* = 3.1, Table 2).

The bystander role yielded the strongest association with age group, *χ* ^2^ (2) = 362.47, *p* < .001, with an effect size of Cramér's *V* of .594 (strong effect). Similar to the defender and pro‐bully roles, the standardized residual analysis indicated that preadolescents were significantly more likely to omit the bystander role (*ASR* = 10.4), whereas adults were significantly more likely to correctly identify it (*ASR* = 8.1, Table 2).

## DISCUSSION

This study aimed to examine how preadolescents and adults observed and interpreted the roles involved in bullying situations. By combining implicit measures (attentional indices) with explicit measures (verbal descriptions), the research provides a comprehensive view of both automatic and conscious cognitive processes in exploring bullying situations. Our findings revealed commonalities between preadolescents and adults, as well as some specific differences.

One key commonality concerned the centrality of the bully and the victim. All attentional indices indicated that these roles attract the most attention of preadolescents and adults, even if they did not have higher visual salience compared to the other roles nor were they positioned more centrally within the scenes. This heightened attention aligned with verbal reports, where the bully and the victim were strongly identified by both groups. The results confirm the centrality of the bully–victim dynamic, aligning with previous studies using self‐report questionnaires (e.g., Guarini et al., 2019), drawings (Bosacki et al., 2006), and eye‐tracking methods (Menabò et al., 2025). This study, however, is the first to confirm the persistence of this attentional pattern also in adults. The prominence of the bully and the victim in both groups in implicit and explicit measures is likely due to their strong emotional and social relevance, as they are directly involved in the core conflict (Menabò et al., 2025). Indeed, the bully typically displays dominance and satisfaction, while the victim exhibits fear and submission. These are intense and easily recognizable emotions that spontaneously capture attention through bottom‐up mechanisms. Human attention is, in fact, automatically guided by emotional cues (McCay‐Peet et al., 2012), and stimuli such as aggressive actions or fearful expressions are particularly salient (Vuilleumier et al., 2001). At the same time, these roles also carry a clear and immediately interpretable social meaning, which explains why the roles of bully and victim consistently stand out across all age groups, with no substantial differences between preadolescents and adults at either the implicit or explicit level.

Differences between groups become evident for secondary roles (defender, pro‐bully, bystanders), both in implicit and explicit measures. Indeed, adults spent significantly more time fixating on the defender, the pro‐bully, and the bystander compared to younger participants. They also revisited the defender and the pro‐bully more frequently, as indicated by higher visit counts, and explored them in greater depth, as shown by the higher number of fixations. At the same time, adults were better at identifying and describing all three secondary roles.

The differences observed can be explained by the interaction between the development of more sophisticated attentional mechanisms and environmental experiences. Attention arises from the combination of bottom‐up and top‐down processes, which together give rise to an ‘integrated salience map’ (Treue, 2003), where the perceptual strength of a stimulus is combined with its behavioural relevance. With age, top‐down control, which develops in parallel with cognitive maturation (e.g., Quadflieg & Penton‐Voak, 2017), becomes progressively more precise and effective, and this may explain the differences found in our results. At the same time, interpersonal experiences further enrich attentional mechanisms by providing more complex interpretive schemas. From this perspective, adults can be considered true ‘experts’ in social interactions, just as in other domains where experience makes visual exploration more efficient and targeted (Gegenfurtner et al., 2011). Cognitive development and social experience therefore reinforce each other, as also highlighted by broader research on social attention, which has shown that adults are indeed more skilled at observing and interpreting social interactions (De Lillo et al., 2021; Mihai, 2022). Together, these two factors support the integration of secondary roles into the overall representation of the scene, enriching the global understanding of bullying situations. This complementarity between cognitive development and social experience was also evident in the explicit measures, as revealed by verbal reports. This result reflects the constructivist view that language develops through the interaction between cognitive maturation and life experiences (Rowland et al., 2025), allowing for more elaborate descriptions of social roles.

Following the discussion of the results concerning secondary roles in general, the next paragraph offers specific considerations regarding each of the three roles examined.

A particularly notable finding was the consistency between implicit and explicit measures for the defender and the pro‐bully, suggesting that these roles hold greater relevance for adults, likely because they played a crucial role in shaping the outcome of a bullying situation. The defender has the power to interrupt the aggression, while the pro‐bully reinforces and amplifies the bully's behaviour, escalating the conflict (Gini et al., 2021; Salmivalli et al., 1996). The fact that all attentional indices were significant further supports this interpretation: adults not only spent more time compared to preadolescents in fixating on defenders and pro‐bullying but also explored multiple areas of interest within them, likely comparing their actions and reactions to those of other figures in the scene.

The bystander role followed a partially different pattern. On the one hand, the number of visits and fixations was similar across age groups, with the only significant difference observed in the total fixation duration. On the other hand, the bystander role had the greatest difference in verbal recognition between adults and preadolescents. This discrepancy suggests that while younger participants visually register the presence of the bystanders, they fixate on them for a shorter time and struggle to interpret their role or express it verbally. In contrast, adults naturally integrate their understanding of this role into their descriptions. A possible explanation is that the bystander's role appears more ambiguous to preadolescents, whereas adults, having more experience with complex social dynamics, recognize it more clearly. Preadolescents, indeed, may find it difficult to assign a precise function to the bystander, reflecting a developmental progression in their ability to articulate complex social interactions. These findings indicate that although the bystander is visually noticed across age groups, the cognitive and verbal elaboration of their role evolves with age and social maturity.

### Limits and future research

While this study provides valuable insights into attentional patterns in bullying scenarios across different age groups, it has some limitations. First, although we examined differences between preadolescents and adults, we did not include younger children, late adolescents, or older adults, leaving an unexplored developmental window. Expanding the age range could reveal when attentional biases towards bullying roles first emerge, how they change across adolescence, and whether they shift in later adulthood, particularly in light of evolving social priorities and cognitive resources. Future research should therefore adopt a broader developmental approach, capturing both early and late stages of social attentional development and examining within‐group differences during key life‐span transitions.

Second, we lacked detailed information regarding participants' educational backgrounds, SES, and professions within the adult group. It is plausible that individuals with different experiences might interpret bullying scenarios differently. Moreover, our adult sample was unbalanced with more female participants, which could further influence attentional and interpretative patterns. Professionals who regularly engage with social dynamics, such as educators, psychologists, or teachers, might allocate attention more strategically and form more complex representations of bullying interactions compared to those in fields with less emphasis on social cognition. Additionally, females and males may have different experiences and perspectives on bullying, potentially shaping their attentional and interpretative strategies in distinct ways. Future studies with larger samples should consider the role of professional background, as well as other socio‐demographic variables, in shaping attentional and interpretative strategies in bullying contexts, as well as possible differences in the function of gender.

A further consideration concerns differences in recruitment procedures between age groups. While participation was voluntary in both cases, adults were recruited through personal networks, which may involve a greater degree of self‐selection compared to the youth sample, potentially influencing attentional and interpretive patterns. By contrast, preadolescents were recruited at school, involving the whole class. Future research should, where possible, adopt parallel recruitment strategies.

Moreover, consistent with prior research showing that adults generally display greater attentional capacity and more refined social processing than younger individuals, our study extends this evidence to the specific domain of bullying, an emotionally charged and socially complex interaction. However, because we only included bullying‐related vignettes, we cannot determine whether the observed differences were specific to bullying or reflected broader, context‐independent developmental patterns in social attention. Future research should include control scenarios to assess either the specificity of our findings to bullying situations or their generalization to other social contexts.

We also did not assess other factors that could influence gaze allocation. Prior research has shown that previous experiences with bullying can shape attentional patterns (e.g., Menabò et al., 2024), while broader work suggests that individual differences such as aggressiveness (Horsley et al., 2010), optimism or pessimism (Isaacowitz, 2005), and emotion regulation strategies (Nummenmaa et al., 2009) may also modulate visual attention. Including these measures would help clarify how these factors interact with attentional processing and, potentially, with the behavioural strategies individuals adopt in real‐world situations. In addition, controlling for them might even reduce apparent gaps between adults and children, isolating the role of age‐related cognitive development.

Finally, a methodological limitation concerns the sequence of tasks adopted. In line with Mihai (2022), we first asked participants to observe the scenes freely, and only afterwards to describe them verbally, in order to capture spontaneous attentional allocation without introducing possible priming effects. However, it is possible that the order of these tasks shaped how participants processed the scenes. For example, if verbalization had been required before or simultaneously with the eye‐tracking task, attentional patterns might have been altered. Although we considered this sequence necessary to avoid conditioning spontaneous attention, future studies could profitably manipulate task order and examine whether correlations between eye‐tracking and narrative data vary under different procedural arrangements.

### Implications and conclusions

The findings of this study have significant implications for educational practices and interventions. While both preadolescents and adults naturally focus on the bully–victim dynamic, adults possess a broader understanding of bullying scenes, including the role of secondary figures. Such a perspective can be leveraged to design targeted tools, resources, and strategies aimed at helping preadolescents move beyond an exclusive focus on the most salient cues and towards a richer, more integrated view of social dynamics. In practice, this could include the development of classroom activities that train students to recognize and discuss the roles of defenders, pro‐bullies, and bystanders, for example, through guided discussions where teachers prompt students to consider the perspective of each role (Guarini et al., 2019). Interactive storytelling and digital narratives can also be used to illustrate how different roles influence the course of bullying episodes and to stimulate collective reflection (Cordi & Masturzo, 2013). Role‐playing scenarios, whether in traditional classroom settings or through virtual reality environments, offer repeated opportunities to experience and reflect on complex social interactions (Oyekoya et al., 2021), encouraging students to experiment with alternative responses and to increase their awareness of the impact of each role. By engaging in these activities, students can move from a bottom‐up approach, where their understanding is primarily driven by immediate, salient cues, to a top‐down approach, where accumulated experiences, learned interpretive ‘lenses’, and deliberate attention control shape social understanding, following the SIP model, which emphasizes how experiences can modify the steps of social information processing. Importantly, these abilities can be nurtured from childhood, thus strengthening children's capacity to interpret subtle relational cues, respond constructively in real‐life situations, and potentially transform challenging past experiences, such as prior victimization, into empathy, resilience, and prosocial skills.

In conclusion, to our knowledge, this is the first study to combine eye‐tracking data with verbal reports to investigate how attention to bullying roles varied between preadolescents and adults. The results showed that the bully and the victim received the most attention across all age groups, reinforcing their centrality in bullying dynamics. However, adults demonstrated greater sensitivity to the other roles: defenders, pro‐bullies, and bystanders, devoting more attention to them and describing them with greater accuracy. This suggested that improvements in attentional control with age facilitated a more detailed recognition of roles and that social experience played a key role in organizing and verbalizing social information. By leveraging adults' enhanced ability to process social cues, educators and policymakers can create environments that promote more comprehensive social awareness and positive peer interactions.

## AUTHOR CONTRIBUTIONS

**Laura Menabò:** Conceptualization; formal analysis; methodology; data curation; investigation; writing – original draft; writing – review and editing. **Annalisa Guarini:** Conceptualization; supervision; methodology; investigation; writing – review and editing; data curation.

## FUNDING INFORMATION

No specific funding was received.

## CONFLICT OF INTEREST STATEMENT

The authors report no conflict of interest.

## Data Availability

The data that support the findings of this study are available from the corresponding author upon reasonable request.
